# Accurate prediction of birth implementing a statistical model through the determination of steroid hormones in saliva

**DOI:** 10.1038/s41598-021-84924-0

**Published:** 2021-03-10

**Authors:** Silvia Alonso, Sara Cáceres, Daniel Vélez, Luis Sanz, Gema Silvan, Maria Jose Illera, Juan Carlos Illera

**Affiliations:** 1 Department of Physiology, School of Veterinary Medicine, University Complutense of Madrid, 28040 Madrid, Spain; 2 Department of Statistics and Operational Research, Faculty of Mathematics, University Complutense of Madrid, 28040 Madrid, Spain

**Keywords:** Computational biology and bioinformatics, Machine learning, Endocrine system and metabolic diseases

## Abstract

Steroidal hormone interaction in pregnancy is crucial for adequate fetal evolution and preparation for childbirth and extrauterine life. Estrone sulphate, estriol, progesterone and cortisol play important roles in the initiation of labour mechanism at the start of contractions and cervical effacement. However, their interaction remains uncertain. Although several studies regarding the hormonal mechanism of labour have been reported, the prediction of date of birth remains a challenge. In this study, we present for the first time machine learning algorithms for the prediction of whether spontaneous labour will occur from week 37 onwards. Estrone sulphate, estriol, progesterone and cortisol were analysed in saliva samples collected from 106 pregnant women since week 34 by enzyme-immunoassay (EIA) techniques. We compared a random forest model with a traditional logistic regression over a dataset constructed with the values observed of these measures. We observed that the results, evaluated in terms of accuracy and area under the curve (AUC) metrics, are sensibly better in the random forest model. For this reason, we consider that machine learning methods contribute in an important way to the obstetric practice.

## Introduction

In the obstetric field, one of the main objectives is the understanding of the specific physiology at the beginning of parturition and the hormonal interaction, as these concepts are crucial for the adaptation of the fetus to extrauterine life and labour.

The gestation period is considered at term between week 37–42^1^. The perinatal mortality rate increases from 2 to 3% in week 40 to 4–7% in week 42^2^, with post-term pregnancy a reason for the induction of labour^3^. Currently, there is no set time for induction, although it is recommended between week 41–42^4^. Only 4% of infants are born on the estimated date of delivery, which is calculated based on the date of the last menstruation plus 280 days, with an error of two weeks^5^. Childbirth involves the complex relationship between mother, fetus and placenta that implies a complex interaction of biomolecular, immunological and endocrine mechanisms, modulated by aetiology, ethnicity and gestational age^6^. It is a perfect coordination of events that include progressive effacement and dilation of the cervix, rupture of the amniotic membranes, and initiation and maintenance of effective uterine contractions, culminating in labour^7^.

The development of pregnancy is under hormonal control of the fetoplacental unit. Progestogens, estrogens, androgens and glucocorticoids are secreted during pregnancy and their interaction modulates different cellular and physiological mechanisms^8^.

Estrogen concentrations progressively increase during pregnancy^9^ and are involved in the increase of the size of the uterus, the stimulation of the expression of oxytocin (OT) and progesterone (P4) receptors and the induction of synthesis of hepatic proteins. In late pregnancy, estrogens are involved in the activation of the labour mechanism^8^.

Estriol (E3) is the dominant estrogen during pregnancy. It is produced by the placenta from dehydroepiandrosterone sulphate (DHEAS) synthesised exclusively by the fetal adrenal gland, and is used as an indicator of the function of the fetoplacental unit^10–12^. Another important estrogen during pregnancy is estrone sulfate (E1SO4), which acts as a reserve for the peripheral formation of bioactive estrogenic forms^13^.

In comparison, P4 is essential for the maintenance of pregnancy as it inhibits the contractility of the myometrium by decreasing the production of prostaglandins and the genetic expression of contraction associated proteins (CAPs)^14^.

Regarding glucocorticoids, cortisol (C) is of vital importance in pregnancy, since it is responsible for fetal lung maturation^15^. The increase in C levels at the end of pregnancy can serve as a signal for the fetus to induce estrogen synthesis prior to the onset of delivery^16, 17^.

The endocrine system is a complex regulatory system with multiple levels of organisation (central nervous system, tissues, adrenal glands, cells) and time cycles (circadian oscillations, rapid responses, ultradian rhythms). This system displays nonlinear feedback responses involved in the interaction between its components and other parts of the body^18^. Statistical models have assisted in understanding how feedback mechanisms are key to homeostatic modulation^18^, and also the behaviour of the complex pituitary-adrenal–pituitary axis complex^19^.

Traditional logistic regression models are generally used to face a problem of predicting premature births^20^. However, the use of machine learning models has aroused growing interest and have been widely employed in bioinformatics^21^, as well as in different fields of health sciences^22, 23^. The possibility of its use is also mentioned in the diagnosis of premature births and emerging obstetric diseases^20^. In this way, machine learning models, such as tree-based algorithms and neural networks, can be used to address problems related to the prediction of labour. In this study, random forest models were used to contrast the contribution of such a machine learning technique, compared to traditional logistic regression models.

Therefore, the aim of our study was to determine P4, C, E3 and E1SO4 levels by a non-invasive method, in saliva samples from healthy pregnant women during the third trimester of pregnancy (from week 27 to the time of delivery), in order to develop a mathematical model to predict the probability of a spontaneous birth.

## Results

### Validation parameters

The sensitivity of the EIA technique was tested by means of low detection limit and calculated in 10 consecutive assays. Results were P4 = 0.25 nmol/L, C = 0.14 nmol/L, E3 = 0.13 nmol/L and E1SO4 = 0.31 nmol/L. The accuracy of the EIA was tested by determining the recovery rates of known amounts of P4, C, E3 and E1SO4 spiked into saliva samples. The average range of recovery rates was as follows (mean ± standard deviation): P4 = 94.29 ± 3.85 to 98.71 ± 5.37; C = 94.29 ± 3.85 to 97.15 ± 2.68; E3 = 90.75 ± 3.92 to 99.65 ± 2.01; and E1SO4 = 90.08 ± 3.29 to 93.27 ± 0.90. Precision of P4, C, E3 and E1SO4 was determined by calculating the intra- and inter-assay coefficients of variation (CV%). P4 = intra-assay: 3.2 ± 0.98%, inter-assay: 5.3 ± 0.89%; C = intra-assay: 4.8 ± 1.02%, inter-assay: 7.4 ± 1.23%; E3 = intra-assay: 6.7 ± 1.32%, inter-assay: 9.1 ± 2.14%; and E1SO4 = intra-assay: 2.9 ± 0.84%, inter-assay: 6.5 ± 1.36%. In order to determine the effects of saliva on standard curve, the standard curves with saliva samples were run in parallel with the standard dose–response curve. There was a good degree of parallelism between both standard curves for the hormones studied: P4, R = 0.87; C, R = 0.84; E3, R = 0.89; and E1SO4, R = 0.83.

### Hormonal concentrations

**Figure 1:**
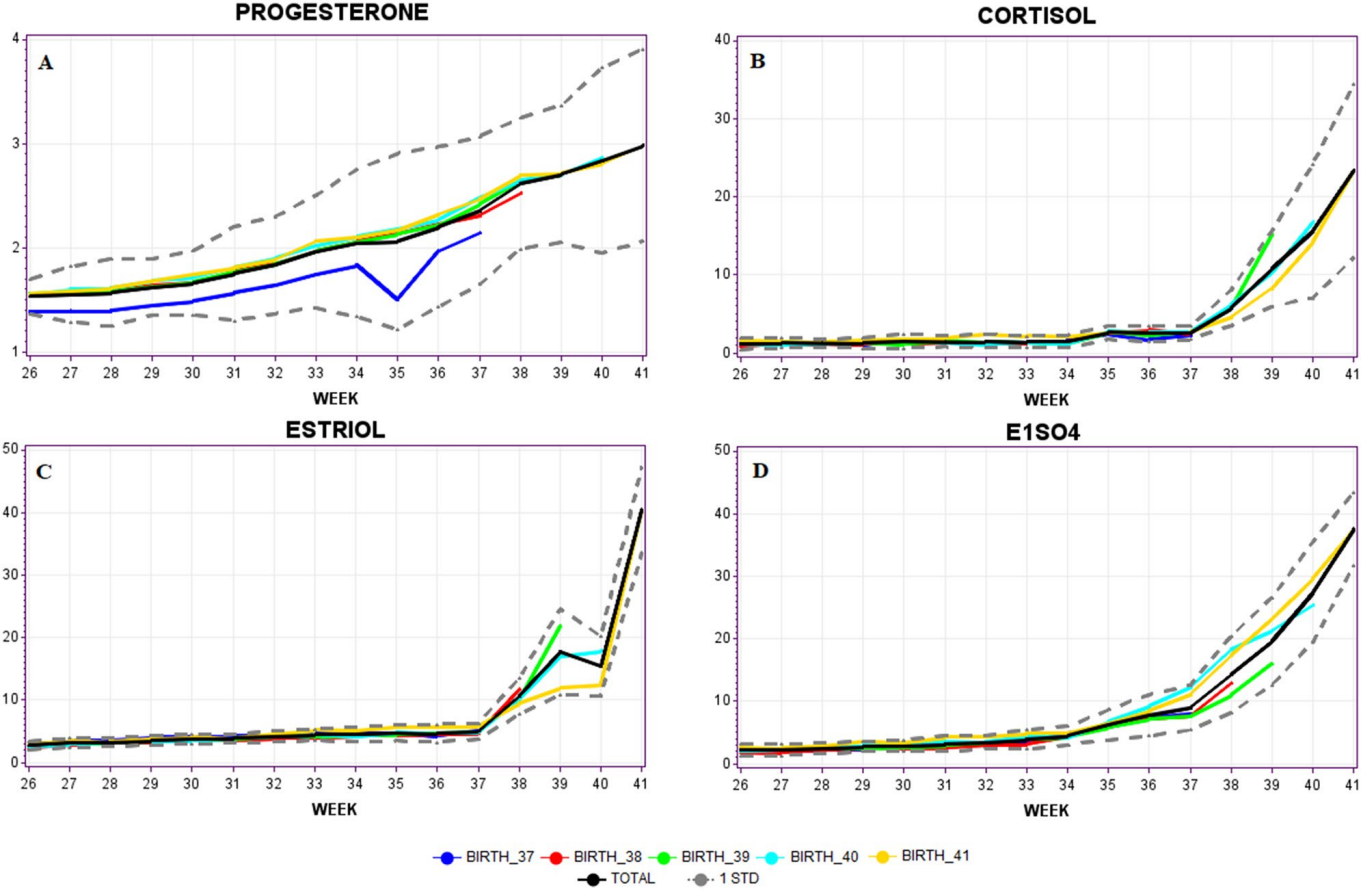
Progesterone (**A**), cortisol (**B**), estriol (**C**) and estrone sulphate (**D**) mean concentrations during third trimester of pregnancy (from 34th week until delivery). Values corresponded to each group of women, distinguished by the week of delivery.

Total means of each hormone together with a confidence interval based on one standard deviation were calculated from week 26–41 (Fig. 1 and supplementary Table 1). In addition, the evolution of mean values distinguished by the week of delivery is also shown. Among all the hormones analysed, P4 is the only one that increased gradually throughout the weeks of pregnancy during the third trimester (Fig. 1A). C and E3 results showed that concentrations increased significantly (*p* < 0.05) from week 37 until giving birth (Fig. 1B,C). E1SO4 concentrations started increased exponentially from the 35^th^ week until giving birth (Fig. 1D).

**Table 1:** Repeated measures correlation (rmcorr) of P4, C, E3, and E1SO4 concentrations in third trimester of gestation.

From 26–37 weeks		
$v1$	$v2$	$\rho_{v1v2}$
CORTISOL	ESTRIOL	0.374
CORTISOL	PROGEST	0.542
CORTISOL	E1SO4	0.408
ESTRIOL	PROGEST	0.251
ESTRIOL	E1SO4	0.365
PROGESTE	E1SO4	0.314

Regarding repeated measures correlation coefficient (rmcorr) between hormones, results demonstrated that all hormones are positively correlated (Table 1), with C as the one presenting the strongest correlation with the rest of the hormones while P4 as the one with the weakest correlation.

### Statistical model

We have adjusted the statistical models in order to predict the probability of spontaneous birth in the following week, from gestation week 37 (37th week included), based on the hormonal levels collected since the 34th week (Fig. 1). It was decided to adjust two types of statistical models. On the one hand, we fit a multivariate logistic regression model, since it is a reference model in the biosanitary field, mainly due to its simple interpretation. Such a model allows the measurement of the influence on the response variable of the independent variables by means of the odds ratio associated with each of the estimated parameters. On the other hand, a random forest model has been adjusted, which is a model that loses the interpretability of the results, but in return, is very competitive from the predictive point of view^25^.

Both models have been implemented using SAS Institute Enterprise Miner software, which internally invokes the DMREG procedure to perform the logistic regression and HPFOREST procedure to perform the random forest, proposing accuracy as the metric to be maximised.

In the logistic regression model, the logit function has been considered as the linkage function. Also, a stepwise variable selection process has been used, proposing a significance value of 0.05 for the sl-entry and sl-stay parameter. To avoid possible overfitting effects in the adjustment of the models, we have applied a cross-validation strategy. The value obtained for the accuracy metric in this process have been 77.4%. The assessment over the test dataset has provided an accuracy of 69.07% (Table 2).

**Table 2:** Confusion matrices of logistic regression and random forest models over test dataset.

Logistic regression				Random forest			
Actual	0	1	Total	Actual	0	1	Total
**Predicted**							
0	TN = 52	FN = 15	67	0	60	13	73
1	FP = 15	TP = 15	30	1	7	17	24
TOTAL	67	30	97	TOTAL	67	30	97
Accuracy	69.07%			Accuracy	79.38%		
PPV	50.00%			PPV	70.83%		
NPV	77.61%			NPV	82.19%		
AUC	0.707			AUC	0.819		

TN = number of true negative cases, FN = number of false negative cases, FP = number of false positive cases, TP = number of true positive cases, PPV = positive predictive value (precision), NPV = negative predictive value, AUC = area under the ROC.

Being:$$Accuracy = \frac{TN + TP}{{TN + FN + FP + TP}}$$, $$PPV = Positive Predictive Value \left( {Precision} \right) = \frac{TP}{{TP + FP}}$$, $$NPV = Negative Predictive Value = \frac{TN}{{TN + FN}}$$

Table 3 shows the order in which the variables have been included in the model. No variables were removed in the adjustment process.

**Table 3:** Evolution of training success rate and cross validation of stepwise logistic regression.

Step	Sense	Variable	Cross-validation accuracy (%)
1	Entry	ESTRIOL_VAR_WEEK	76.41
2	Entry	CORTISOL_FORTNIGHT	74.87
3	Entry	E1SO4_VAR_FORTNIGHT	76.92
4	Entra	PRIMIPARA	73.33
5	Entry	AGE	76.92
6	Entry	PROGESTERONA_MONTH	77.44

Table 4 shows the estimates of the model coefficients, the corresponding p-values and the odds ratios associated with variables selected.

**Table 4:** Estimation, p-values and odds ratios associated with variables of logistic regression model.

Variable	Estimation	SE	p-value	Odds ratio
INTERCEPT	7.1399	2.8555	0.0124	
ESTRIOL_VAR_WEEK	8.9654	1.9565	< 0.0001	999
CORTISOL_FORTNIGHT	0.1987	0.0549	0.0003	1.22
E1SO4_VAR_FORTNIGHT	− 0.9205	0.4175	0.0275	0.398
PRIMIPARA (NO)	0.6204	0.2039	0.0023	NO VS YES (3.458)
AGE	− 0.2045	0.0748	0.0062	0.815
PROGESTERONA_MONTH	− 0.916	0.4646	0.0486	0.4

We can observe that the positive and negative signs of the coefficients associated with the hormonal variables are consistent with the biological functions. We think that negative values for E1SO4 and progesterone are related to longer pregnancies, while positive signs for estriol and cortisol are related to earlier deliveries.

In the random forest model, a maximum of 100 decision trees were considered. Regarding the value of the parameters associated to the trees, following are those considered by default in Enterprise Miner:

- Maximum depth = 50
- Number of training observations a node must have to consider splitting it = 30
- Minimum number of observations per leaf = 1
- Percent of the training dataset randomly selected to adjust each one of the trees = 60%. Seed used for generating random numbers = 12,345
- Number of variables by tree = $int\left( {\sqrt {total\,number\,of\,variables} } \right) = {\text{int}}\left( {\sqrt {22} } \right) = 4.$

In this case, to avoid possible overfitting, we used a modelling strategy used by Enterprise Miner called Out-Of-Bag (OOB) because it is the alternative this software proposed to the well-known cross-validation strategy. In the OOB strategy, the observations associated with each one of the adjusted trees are called the bagged observations, while those excluded are called OOB observations. These last observations were used in order to decide the number of trees to be ensembled to improve the capacity of generalisation of the model, similar to a cross-validation strategy.

**Figure 2:**
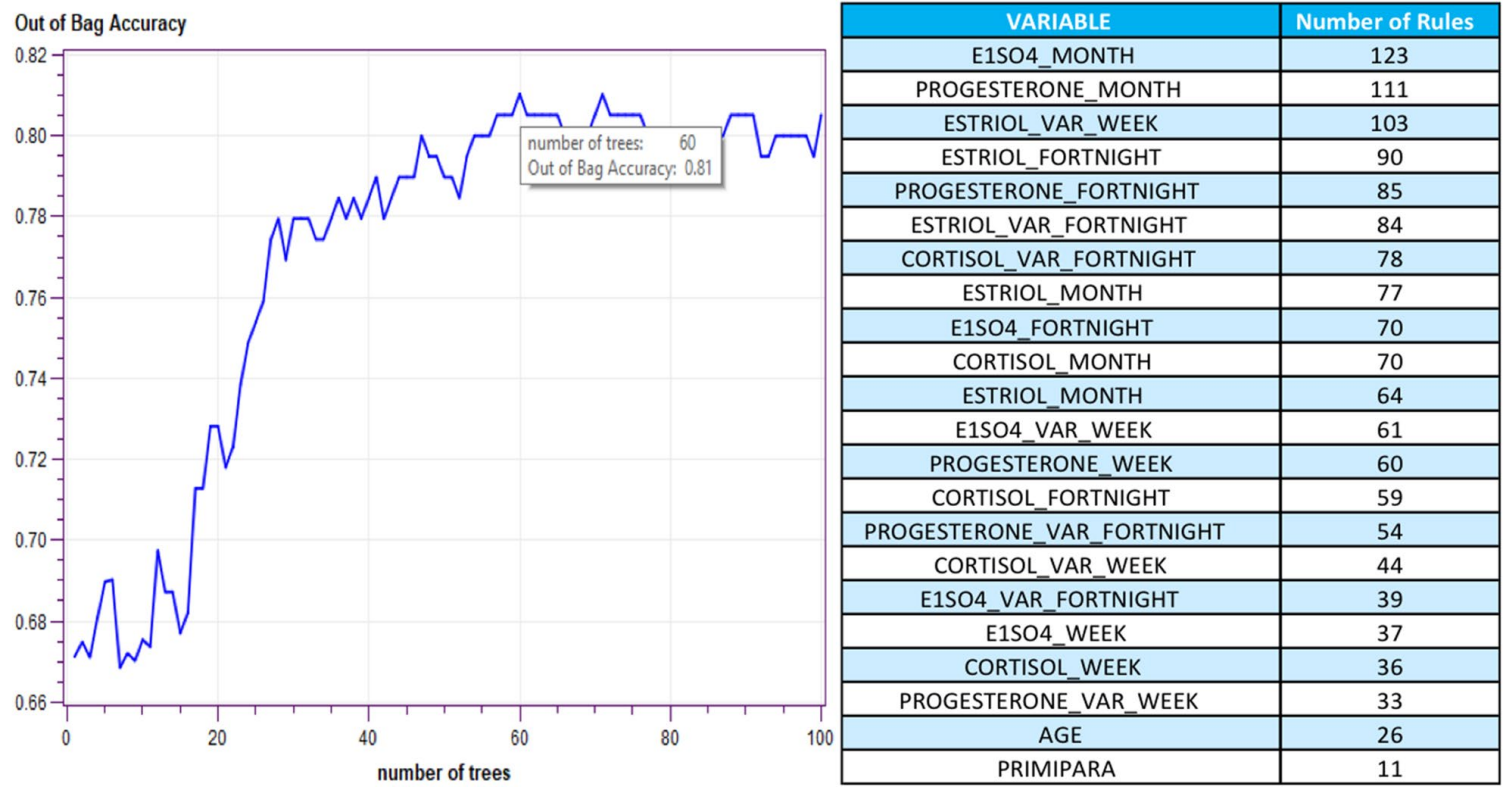
Evolution of success rate in training and Out-Of-Bag samples with random forest model.

The evaluation of the accuracy metric leads us to consider as an optimal model the ones that ensemble a total of 60 or 71 trees (Fig. 2), showing an OOB accuracy of 81.03%. According to the principle of parsimony (Occam’s razor) recommended in the context of modelling^26^, we decided to use the simplest model, therefore, the one covering 60 trees was taken. The assessment over the test dataset provided an accuracy of 79.38% (see Table 2).

A list representing the relative importance of the variables in this model is also shown in Fig. 2. This relevance is established based on the degree of participation of these variables in each of the 60 trees that comprise the model. The fact that the same variable can participate in the same tree several times justifies that the participation of each variable can be greater than 60. Therefore, it can be seen that the E1SO4 value average over the four weeks of the last month (E1SO4_MONTH variable) is the most important effect to be considered. The same average values of Progesterone-PROGESTERONE_MONTH and the percentage variation of Estriol- ESTRIOL_VAR_WEEK over the last two weeks were, respectively, the second and third most relevant variables, being the only ones that participate in more than 100 trees. The first variable associated with cortisol hormone appeared in the seventh position.

In Table 2, the confusion matrices associated with both models are presented. The accuracy, the positive and the negative predictive value (PPV and NPV, respectively) showed that the random forest model provides better results than the logistic model. The area under the Receiver Operating Characteristics (ROC) curve, one of the most important evaluation metrics to verify the performance of any classification model^27^, showed that the random forest model works better also.

Moreover, two criteria were used as a baseline to compare the accuracy of the models. Firstly, the maximum randomness criterion, consisted of assigning the dominant class (birth = 0) to all cases. Although we considered this criterion to be very conservative, it was observed that the reference value it provides is 67/97 (69.07%), which is equal to the accuracy of the logistic regression, but significantly less than that of the random forest model (79.38%). Secondly, the proportional randomness criterion consisted of randomly assigning 0 and 1 values according to the actual proportion of the binary event analysed. Assuming that the predictions are made at random regardless of the real values, the expected probability of coincidences using this criterion is 0.5727 (57.27%).

$${\text{P}}\left( {{\text{actual}} = 1 \cap {\text{predicted}} = 1} \right) + {\text{P}}\left( {{\text{actual}} = 0 \cap {\text{predicted}} = 0} \right) = {\text{ p}}^{2} + \left( {1 - {\text{p}}} \right)^{2} = \left( {\frac{67}{{97}}} \right)^{2} + \left( {\frac{30}{{97}}} \right)^{2} = 0,5727$$

being $${\text{p}} = {\text{P}}\left( {{\text{actualBirth}} = 1} \right)$$ and $$1 - {\text{p}} = {\text{P}}\left( {{\text{actualBirth}} = 0} \right)$$.

The second criterion is considered reasonable to test the true goodness of fit of the model. Then, in the case of the multivariate logistic regression model, the reference precision improves by 11.80 percentage points (69.07–57.27%), while with the random forest model the improvement is 22.11 percentage points (79.38–57.27%) (Table 5).

**Table 5:** Confusion matrices evaluated per week on test table by random forest model.

WEEK	37			38			39			40		
Actual	0	1	OVERALL	0	1	OVERALL	0	1	OVERALL	0	1	OVERALL
**Predicted**												
0	29	4	33	17	5	22	11	3	14	3	1	4
1	1	1	2	3	5	8	1	5	6	2	6	8
OVERALL	30	5	35	20	10	30	12	8	20	5	7	12
Accuracy	85.71%			73.33%			80.00%			75.00%		
PPV	50.00%			62.50%			83.33%			75.00%		
NPV	87.88%			77.27%			78.57%			75.00%		

TN = number of true negative cases, FN = number of false negative cases, FP = number of false positive cases, TP = number of true positive cases, PPV = positive predictive value (precision), NPV = negative predictive value, AUC = area under the ROC.

These results led us to propose the random forest model as appropriate to predict the week in which birth is most likely to occur, although it has less explanatory power than the logistic regression model.

Finally, the confusion matrices, detailed per week, are presented in Table 5. It was observed that the accuracy of the model is higher in the 37th week (85.71%), but this result must be interpreted carefully because in this week, the model tends to predict birth = 0, taking into account that it is the week in which fewer births occur. It should be considered that this prediction was provided by the model in 33 of the 35 cases, and hence in almost 94.30% of the cases. This allowed the highest NPV value (87.88%) but, this should not be considered as an important result because the maximum randomness criterion would provide a value of 85.71% (30/35). On the other hand, the number of positive cases predicted in each week was similar to the actual cases in the weeks numbered 38, 39, 40 (eight vs. 10, six vs, eight, eight vs. seven, respectively) and therefore, the corresponding results could be considered more interesting.

## Discussion

In this study, the physiological variations of P4, C, E3 and E1SO4 during the third trimester are shown. These variations played an important role in pregnancy and in the preparation of labour mechanisms. It is convenient to explain that the activation of labour was not directly related to changes in the levels of estrogen and progesterone; however, this event could be affected by an alteration in the response of the myometrium to the expression of estrogen receptors (ER) and progesterone receptors (PR). The interaction between PR and ER of the myometrium prevents the action of estrogens during most of the pregnancy, while in preparation for delivery, there is a withdrawal of progesterone and estrogen activation^28^.

We implemented the analysis of these hormones in saliva samples, a useful non-invasive method to predict the week of spontaneous delivery. Our results revealed a positive hormonal correlation between all studied hormones, with C being the strongest correlation to the rest of hormones. Proper functioning and interaction of steroidogenic tissues, in addition to the enzymes involved in their synthesis and transport, are crucial since intrauterine exposure of the fetus to abnormal glucocorticoid levels^29^ or sex hormones^30, 31^ can negatively affect fetal development. Some authors state that there is a relationship between fetal sex with estrone and estradiol levels during pregnancy, however, they did not find statistically significant results^12^.

The role of the different sex hormones has been extensively studied, although there are hardly any articles that interrelate these levels of estrogens, progestogens and glucocorticoids and their variations throughout the third trimester of pregnancy. According to the results obtained in this study, the hormonal analysis of P4, E3, C and E1SO4 at specific times of the third trimester were good indicators of normal values in low-risk pregnancies, and their determination could provide additional information relevant to the control of pregnancy.

In addition, the exponential increase in P4, C, E3 and E1SO4 in third trimester, the period in which the fetus reaches maturity^32^, prepares the fetus and mother for the start of labour, as reported by other authors^8, 33–35^. Although its biological importance is ambiguous, E1SO4 seems to serve as an estrogenic reservoir linked to the endometrium transformation of the endometrium necessary for pregnancy maintenance^36^. The increase in E1SO4 at week 35 determines the increases in the other hormones studied. Therefore, it is critical to take this measurement last during the third trimester of pregnancy, in order to obtain a better understanding of the onset of labour processes.

The relevance of P4, C, E3 and E1SO4 in pregnancy and the onset of labour led us to develop a statistical model that could predict the week of birth. In recent years, the need to develop new prognostic methods in the field of obstetrics in order to improve clinical practice regarding the prediction of premature births, delivery methods, and development of pre-eclampsia, among others, has been emphasised^37^.

The knowledge of the week of spontaneous birth one week in advance has multiple benefits for both the pregnant woman and the health personnel, and has been very useful for reducing the number of inductions to labour for post term pregnancies. The development of a mathematical model with a positive predictive value of 70.83%, as well as a negative predictive value of 82.19%, was capable to relate from a woman the variations of the different hormones of only four saliva samples collected from week 34 and to predict childbirth. Therefore, this model is a novel and great advance in the field of obstetrics.

The application of the statistical model developed in low-risk pregnant women, routinely from week 34, contributed to improving the allocation of health resources based on the number of expected deliveries, as an increase in the number of midwives and obstetricians is associated with a decrease in the cesarean section ratio^38^. Also, its routine application contributed to reducing the number of inductions of labour (IOL) for post-term pregnancy, confirming that labour will begin the week following the application of the statistical model. This action could specify the maximum period to maintain a pregnancy, giving the opportunity of a spontaneous delivery and reducing the complications associated with IOL as postpartum haemorrhage, uterine rupture^39^, instrumental deliveries, cesarean sections and used of epidural anaesthesia^40, 41^, among others.

For future research, among other aspects, the inclusion of criteria such as pre-pregnancy body mass index, previous preterm birth or history of progesterone medication, would enhance the results of future randomised forest models^45^.

Therefore, due to the development of a statistical model that predicts the due date in low-risk women based on the levels of P4, SO4E1, E3 and C from week 34, fetal survival, the allocation of health resources based on the number of expected delivery, as well as the reduction of the number of inductions to post-term delivery, could be markedly improved.

## Methods

### Sample collection

This study was carried out at the Nuevo Belén Clinical University Hospital (Madrid, Spain), in collaboration with the Department of Animal Physiology of the Complutense University of Madrid (Spain). The process of patient recruitment and sample collection was carried out for over a year. An informed consent was obtained from all participants recruited on this study.

A total of 161 healthy pregnant women aged between 27 and 44 years (34.88 ± 3.29) were recruited (data from all recruited woman are summarised in Table 6), of which two of the initial participants dropped out before the beginning of the study. We therefore commenced with a total of 159 women, 53 of whom dropped out throughout the study, reaching a total of 106 who completed the study. The exclusion criteria were kidney disease, thyroid disease, autoimmune disease, cancer, pregestational diabetes, pre-gestational hypertension, in vitro fertilisation with heparin treatment, and current steroid treatment.

**Table 6:** Data for all recruited women.

	All participants recruited
	n = 161
	Participants completed study
	n = 106 (Dropout rate = 34.2% )
Mean age	34.88 ± 3.29
**Prepregnancy BMI (n, %)**	
Normal weight	97 (91.5%)
Underweight	9 (8.4%)
**Parity (n, %)**	
Primiparous	66 (62.2%)
Multiparous	40 (37.7%)
**Current pregnancy**	
Mean gestational age	38 weeks + 6 days
Birth at 37 weeks	17 (16%)
Birth at 38 weeks	30 (28.3%)
Birth at 39 weeks	24 (22.6%)
Birth at 40 weeks	21 (19.8%)
Birth at 41 weeks	16 (15%)
**Mode of delivery**	
Spontaneous birth	63 (59.4%)
Induction of labour (post dates + Spontaneous ruptured of membranes* + other reasons)	43 (40.5%)
Induction of labour for post dates (> 41 + 4)	6 (6.3%)
Vaginal birth	83 (78.3%)
Cesarean delivery- without trial of labour**	9 (8.4%)
Cesarean delivery- after trial of labour	14 (13.2%)
**Rupture of membranes**	
> 24hours	31 (29.2%)
< 24hours	75 (70.8%)
**Newborn**	
Mean birth weight (g)	3479 ± 356gr
**Apgar score < 7**	
One minute	8 (7.5%)
Five minutes	4 (3.7%)
10 min	2 (1.8%)

All indicators were applied according to the SEGO recommendations.

*Spontaneous rupture of membranes was considered spontaneous birth as between 60–80% of births occur within 24 h after SROM and approximately 90% occur by 48 h, however, due to risk of neonatal infection, all study participants were induced 12 h post SROM, as per hospital protocol.

**Cesarean delivery without trial of labour was considered as those performed during latent phase of labour without reaching active labour. Elective cesarean section cases were discarded from the total samples.

Eligible women underwent weekly collection of a saliva sample during the third trimester of pregnancy (from week 27 until week of delivery), with a Salivette (Sarstedt, Germany) collection tube. All samples were collected at an established time (10:00 am ± 1 h).

This study was approved by the Clinical Research Ethics Committee of Hospitales de Madrid, with the code CEIm HM hospitals: 16.06.0960-GHM, in accordance with the World Medical Association and the Declaration of Helsinki.

### Hormone determinations

The Salivette tubes were centrifuged for 15 min at 2000×*g* and 4° C. The saliva obtained was stored at a temperature of − 20 °C until further hormonal analysis. Polyclonal antisera raised in male rabbits against: 11α-hydroxyprogesterone 11α hemisuccinate BSA (P4), estrone-3-glucuronide: BSA (SO4E1), cortisol 3-CMO: BSA (cortisol) and estriol -6-CMO: BSA (Estriol) were used for assay development (Steraloids. Inc., Newport, RI). P4 (ab: C914), E1SO4 (ab: R522-2), E3 (ab: R4835) and C (ab: R4866) concentrations were assayed by enzyme-immunoassay (EIA).

Briefly, microtiter plates (96-well flat-bottom polystyrene) were coated overnight at 4° C with the appropriate antibody dilutions. Then, plates were washed 3 times and conjugate working solutions (CWS) were prepared by diluting conjugate stocks in assay buffer. Standards and saliva samples were diluted in CWS and analyzed in duplicate. To achieve a competitive reaction, plates were incubated at room temperature for 2 h. Plates were washed 3 times with wash buffer and Enhance K-Blue TMB substrate (Neogen, Lexington, KY) was added to each well. To stop colorimetric reaction, stop solution was added to each well. Absorbance was read at 450 nm in an automatic microplate reader. Hormone concentrations were calculated by means of software developed for this technique (ELISA AID, Eurogenetics, Belgium). A standard dose–response curve was constructed by plotting the binding percent-age (B/B0 × 100) against steroid hormone standard concentrations. P4, E3, SO4E1 and C concentrations were expressed in ng/ml^42^.

The validation technique parameters’ recovery rates, sensitivity, intra- and inter-assay coefficients of variation and parallelism were assayed as previously reported by Illera et al.^42^. The EIA techniques and antibodies used were developed and validated in the Endocrinology Laboratory of the Department of Animal Physiology (Faculty of Veterinary Medicine, University Complutense of Madrid, Spain).

### Statistical analysis

In order to achieve the desired precision for the estimates of the population mean hormonal values in each week of observation, the sample size was estimated with a confidence level of 95%, and an error of the estimate of ± 0.2 times the standard deviation, according to this consideration we obtained n ≥ 96.04.

$$\begin{aligned} & Error\,of\,the\,estimate = z_{\alpha /2} \frac{\sigma }{\sqrt n } \\ & z_{\alpha /2} \frac{\sigma }{\sqrt n } \le 0.2\sigma \\ & \alpha = 0.05 \\ & n \ge \left( {\frac{1.96}{{0.2}}} \right)^{2} = 96.04 \\ \end{aligned}$$

To achieve the required precision and based on previous experience with other studies, an approximate loss between 30 and 40% of women has been estimated throughout the sample collection process (in the worst case, the dropout rate was 40%, leading us to specify n ≥ 161). Finally, after concluding the study, n = 106, which is above the initial consideration resulting in a dropout rate 34.2%.

Descriptive statistical analysis for mean and standard deviation of each hormone based on the week of delivery was performed. Repeated measures correlation coefficient (rmcorr) between hormonal values during the third trimester were estimated using PROC MIXED procedures by SAS^24^.

Validation technique parameters (recovery rates, sensitivity, intra- and inter-assay coefficients of variation) were calculated according to Andreasson et al.^43^. Parallelism was calculated using ANCOVA analysis^44^. Data were expressed as mean ± standard error. In all statistical comparisons, type I error set to maximum of 0.05.

### Methodology for the construction of a childbirth prediction model

The methodology employed for predicting the probability of spontaneous delivery in the week following from the latest hormonal levels observed is presented in this section. This methodology is based on two phases: processing data and adjustment of mathematical model.

#### Data processing

A binary variable has been defined for each pair (Woman, Week), identifying whether a subject gives birth (1) or not (0) each week. This is the variable whose value is to be predicted, and its distribution by weeks is shown in Table 7. This table presents a total of 292 cases, distributed into 202 cases with a target equal to 0 and 90 cases with a target equal to 1. This table is divided into two tables (Table 8), one named “training dataset used to adjust the model” and another one named “test dataset used to check it”. The proportions associated with one and another dataset are 2/3 and 1/3, respectively. The cases assigned to each dataset were selected at random, taking into account that the patients registered in one and the other table must be exclusive.

**Table 7:** Binary variable for women and week that identifies whether the woman gives birth (1) or not (0).

WEEK	37	38	39	40	OVERALL
**Birth**					
0	90	60	36	16	202
1	16	30	24	20	90
Overall	106	90	60	36	292

**Table 8:** Learning dataset and test dataset.

	Training dataset					Test dataset				
WEEK	37	38	39	40	OVERALL	37	38	39	40	OVERALL
**Birth**										
0	60	40	24	11	135	30	20	12	5	67
1	11	20	16	13	60	5	10	8	7	30
Overall	71	60	40	24	195	35	30	20	12	97

Once the target (variable to be predicted) was defined, it was necessary to define the explanatory inputs used to make the prediction. Basically, these variables measured mean values and percentage variations of the hormonal indicator during different time periods. The detail of the definition of these variables is shown in Table 9. Considering that the data was collected on Mondays of each week, the explanatory variables were measured in the four weeks prior to which the probability of delivery was estimated, including the forecast week itself. Therefore, the hormonal data collected at weeks 34, 35, 36, and 37 were used to predict whether the birth would occur at week 37, as well as weeks 38 and 39, and finally the data collected at weeks 37, 38, 39 and 40 were used to predict whether the birth would occur at week 40 or week 41. The resulting table, made up of the pair (Woman, Week), the binary target variable (birth or non-birth) and the 22 explanatory variables, served as input for different statistical models.

**Table 9:** Dictionary of variables.

INDICATOR_WEEK	Reflects the level of the indicator in the week before WE *For example: CORTISOL_WEEK measures the value of C in the week before WE*
INDICATOR_FORTNIGHT	Reflects the average level of the indicator in the fortnight before WE (the average of the indicator in the last two weeks) *For example: ESTRIOL_ FORTNIGHT measures the average of the two E3 values recorded in the two weeks prior to WE*
INDICATOR_MONTH	Reflects the average level of the indicator in the month before WE (the average of the indicator in the last four weeks) *For example: PROGESTERONE_MONTH measures the average of the four P4 values recorded in the four weeks prior to WE*
INDICATOR_VAR_WEEK	Reflects the percentage variation of the indicator between the two weeks prior to WE ${\text{INDICATOR}}\_{\text{VAR}}\_{\text{WEEK }} = \frac{{{\text{INDICATOR}}\_{\text{WEEK}}\_1 - {\text{ INDICATOR}}\_{\text{WEEK}}\_2}}{{{\text{INDICATOR}}\_{\text{WEEK}}\_2}}$
INDICATOR_VAR_ FORTNIGHT	Reflects the percentage variation of the indicator between the last two fortnights before WE ${\text{INDICATOR}}\_{\text{VAR}}\_{\text{FORTNIGHT}} = \frac{{{\text{INDICATOR}}\_{\text{FORTNIGHT}}\_1 - {\text{ INDICATOR}}\_{\text{FORTNIGHT}}\_2}}{{{\text{INDICATOR}}\_{\text{FORTNIGHT}}\_2}}$
AGE	Continuous variable that refers to the age of the woman
PRIMIPARA	Binary variable that identifies whether it is the first time (value 1) or not (value 0) that the woman give birth

WE = week in which the target is evaluated, whether or not the woman gave birth.

## Supplementary Information

Supplementary Information
