# Supplementary material for: Accurate prediction of birth implementing a statistical model through the determination of steroid hormones in saliva
Source: Sci Rep. 2021 Mar 10;11:5617. doi: 10.1038/s41598-021-84924-0 (PMC7970941; doi:10.1038/s41598-021-84924-0)
Supplement: Supplementary file 1 — Supplementary Information [file 41598_2021_84924_MOESM1_ESM.docx]

*Supplementary Table 1: Mean and standard deviation of each of the hormones from week 26 to 41 of gestation, corresponding to each group of women, distinguished by the week of delivery.*

| **HORMONE** | **BIRTH_WEEK** | **METRIC** | **N** | **W_26** | **W_27** | **W_28** | **W_29** | **W_30** | **W_31** | **W_32** | **W_33** | **W_34** | **W_35** | **W_36** | **W_37** | **W_38** | **W_39** | **W_40** | **W_41** |
| --- | --- | --- | --- | --- | --- | --- | --- | --- | --- | --- | --- | --- | --- | --- | --- | --- | --- | --- | --- |
| SO4E1 | TOTAL | MEAN | 106 | 2,1930 | 2,2657 | 2,4719 | 2,7467 | 2,8812 | 3,2437 | 3,4524 | 3,9345 | 4,4916 | 6,2186 | 7,7720 | 8,9899 | 14,2799 | 19,6293 | 27,4513 | 37,5687 |
| SO4E1 | TOTAL | STD | 106 | 0,9153 | 0,9236 | 0,8626 | 0,8034 | 0,8672 | 1,2742 | 1,0711 | 1,5296 | 1,4835 | 2,4166 | 3,3117 | 3,5910 | 6,1362 | 7,0048 | 8,0096 | 5,8905 |
| CORTISOL | TOTAL | MEAN | 106 | 1,2166 | 1,2703 | 1,2687 | 1,2511 | 1,5027 | 1,5039 | 1,5014 | 1,4030 | 1,4922 | 2,6247 | 2,4812 | 2,5665 | 5,7783 | 10,8083 | 15,6253 | 23,3287 |
| CORTISOL | TOTAL | STD | 106 | 0,7437 | 0,6334 | 0,5322 | 0,6341 | 0,9015 | 0,7201 | 0,8708 | 0,7535 | 0,7872 | 0,8936 | 1,0532 | 0,8673 | 2,2809 | 4,8591 | 8,5862 | 11,1092 |
| ESTRIOL | TOTAL | MEAN | 106 | 2,7076 | 3,1580 | 3,2678 | 3,5508 | 3,7774 | 3,8542 | 4,1964 | 4,4265 | 4,5442 | 4,7697 | 4,7022 | 5,0219 | 10,6323 | 17,6991 | 15,4745 | 40,4754 |
| ESTRIOL | TOTAL | STD | 106 | 0,6790 | 0,7318 | 0,6864 | 0,7594 | 0,7847 | 0,7110 | 0,8608 | 0,9387 | 1,1150 | 1,3043 | 1,4363 | 1,2535 | 2,9284 | 6,8898 | 4,7587 | 6,8429 |
| PROGESTE | TOTAL | MEAN | 106 | 1,5301 | 1,5501 | 1,5668 | 1,6210 | 1,6618 | 1,7510 | 1,8334 | 1,9623 | 2,0429 | 2,0610 | 2,1985 | 2,3559 | 2,6179 | 2,7066 | 2,8375 | 2,9827 |
| PROGESTE | TOTAL | STD | 106 | 0,1707 | 0,2669 | 0,3234 | 0,2665 | 0,3062 | 0,4524 | 0,4650 | 0,5395 | 0,7082 | 0,8446 | 0,7689 | 0,7098 | 0,6291 | 0,6582 | 0,8908 | 0,9218 |
| CORTISOL | 37 | MEAN | 16 | 1,2843 | 1,1043 | 1,2825 | 0,9838 | 1,7162 | 1,3529 | 1,4792 | 1,3745 | 1,6250 | 2,4593 | 1,7879 | 2,2600 |  |  |  |  |
| CORTISOL | 37 | STD | 16 | 0,8027 | 0,2741 | 0,4302 | 0,4869 | 0,6391 | 0,6597 | 0,7654 | 0,3295 | 0,6362 | 1,1842 | 1,0106 | 0,7311 |  |  |  |  |
| CORTISOL | 38 | MEAN | 30 | 0,8527 | 1,2028 | 1,1720 | 1,0867 | 1,2150 | 1,3796 | 1,3243 | 1,2735 | 1,4165 | 2,5124 | 2,9548 | 2,4757 | 5,9518 |  |  |  |
| CORTISOL | 38 | STD | 30 | 0,3851 | 0,5348 | 0,4569 | 0,5346 | 0,5134 | 0,6350 | 0,6517 | 0,6727 | 0,5996 | 0,6734 | 1,2155 | 0,8198 | 1,8923 |  |  |  |
| CORTISOL | 39 | MEAN | 24 | 1,3711 | 1,5145 | 1,3387 | 1,3605 | 1,1524 | 1,5853 | 1,3986 | 1,2477 | 1,2280 | 2,6289 | 2,1837 | 2,7358 | 5,8070 | 15,1710 |  |  |
| CORTISOL | 39 | STD | 24 | 0,7071 | 0,8725 | 0,7024 | 0,7832 | 0,6269 | 0,6824 | 0,9420 | 0,6805 | 0,6957 | 0,8865 | 0,7464 | 0,9700 | 1,5787 | 4,3675 |  |  |
| CORTISOL | 40 | MEAN | 20 | 1,0971 | 0,9859 | 1,1613 | 1,2700 | 1,8512 | 1,3686 | 1,1253 | 1,2875 | 1,2127 | 2,8544 | 2,6925 | 2,7131 | 6,2235 | 10,3271 | 16,8519 |  |
| CORTISOL | 40 | STD | 20 | 0,5900 | 0,4504 | 0,5078 | 0,6268 | 1,4279 | 0,7359 | 0,3957 | 0,4010 | 0,5864 | 0,8027 | 0,7905 | 0,7539 | 3,1774 | 3,7901 | 8,1102 |  |
| CORTISOL | 41 | MEAN | 16 | 1,6900 | 1,5754 | 1,4293 | 1,5908 | 1,7785 | 1,9043 | 2,3700 | 2,1809 | 2,0938 | 2,6893 | 2,5900 | 2,6964 | 4,5964 | 8,2764 | 14,2236 | 23,3287 |
| CORTISOL | 41 | STD | 16 | 1,0732 | 0,6755 | 0,4417 | 0,5498 | 0,8625 | 0,8681 | 1,0565 | 1,2482 | 1,0832 | 1,0793 | 1,0677 | 1,1024 | 2,5502 | 4,4627 | 9,1976 | 11,1092 |
| ESTRIOL | 37 | MEAN | 16 | 3,0157 | 3,5529 | 3,6142 | 4,0869 | 4,2185 | 4,2414 | 4,3823 | 4,7973 | 4,6325 | 4,7462 | 4,2647 | 5,1569 |  |  |  |  |
| ESTRIOL | 37 | STD | 16 | 0,5192 | 0,6334 | 0,6982 | 0,5749 | 0,5795 | 0,5341 | 0,4990 | 0,5651 | 0,9065 | 0,7741 | 1,8303 | 0,6732 |  |  |  |  |
| ESTRIOL | 38 | MEAN | 30 | 2,3348 | 2,8048 | 3,2124 | 3,3305 | 3,6804 | 3,6540 | 3,9391 | 4,0257 | 4,3340 | 4,3765 | 4,5830 | 4,6983 | 11,7410 |  |  |  |
| ESTRIOL | 38 | STD | 30 | 0,4488 | 0,4630 | 0,7143 | 0,6452 | 0,8183 | 0,7576 | 0,9649 | 0,7507 | 0,8416 | 1,0723 | 1,0964 | 1,0764 | 3,0639 |  |  |  |
| ESTRIOL | 39 | MEAN | 24 | 2,7622 | 3,1956 | 3,1013 | 3,4835 | 3,6006 | 3,8312 | 4,2452 | 4,1745 | 4,5486 | 4,4716 | 4,6653 | 5,0100 | 10,1285 | 21,9591 |  |  |
| ESTRIOL | 39 | STD | 24 | 0,6273 | 0,7796 | 0,6449 | 0,6670 | 0,6388 | 0,7483 | 0,8212 | 0,7462 | 0,7488 | 1,2583 | 1,2682 | 1,3084 | 3,0710 | 7,6475 |  |  |
| ESTRIOL | 40 | MEAN | 20 | 2,6294 | 2,9953 | 3,2773 | 3,3465 | 3,5106 | 3,8471 | 4,1057 | 4,4863 | 4,1980 | 4,9383 | 4,6725 | 4,8288 | 10,0576 | 16,9147 | 17,8713 |  |
| ESTRIOL | 40 | STD | 20 | 0,8693 | 0,7201 | 0,6445 | 0,8210 | 0,8275 | 0,5589 | 0,6557 | 0,7945 | 1,4226 | 1,1061 | 1,4223 | 1,6080 | 2,6874 | 4,4089 | 4,9702 |  |
| ESTRIOL | 41 | MEAN | 16 | 3,0623 | 3,5731 | 3,3385 | 3,7585 | 4,0677 | 3,8600 | 4,4846 | 5,2375 | 5,1250 | 5,7093 | 5,7500 | 5,8820 | 9,5136 | 11,9571 | 12,5246 | 40,4754 |
| ESTRIOL | 41 | STD | 16 | 0,6888 | 0,8687 | 0,7188 | 0,9278 | 0,8563 | 0,8082 | 1,1454 | 1,4085 | 1,5370 | 1,9133 | 1,5740 | 1,4072 | 1,8455 | 2,1118 | 2,2080 | 6,8429 |
| PROGESTE | 37 | MEAN | 16 | 1,3893 | 1,3940 | 1,4000 | 1,4485 | 1,4915 | 1,5693 | 1,6438 | 1,7473 | 1,8325 | 1,5033 | 1,9664 | 2,1400 |  |  |  |  |
| PROGESTE | 37 | STD | 16 | 0,1125 | 0,2378 | 0,2629 | 0,2217 | 0,2995 | 0,4461 | 0,3525 | 0,4410 | 0,5121 | 0,7636 | 0,4667 | 0,5237 |  |  |  |  |
| PROGESTE | 38 | MEAN | 30 | 1,5618 | 1,5680 | 1,5779 | 1,6376 | 1,6664 | 1,7746 | 1,8509 | 1,9691 | 2,0596 | 2,1288 | 2,2239 | 2,3104 | 2,5265 |  |  |  |
| PROGESTE | 38 | STD | 30 | 0,2115 | 0,2833 | 0,2488 | 0,2691 | 0,2559 | 0,4544 | 0,4960 | 0,5339 | 0,5238 | 0,7470 | 0,8373 | 0,6466 | 0,7142 |  |  |  |
| PROGESTE | 39 | MEAN | 24 | 1,5626 | 1,5745 | 1,5830 | 1,6260 | 1,6706 | 1,7700 | 1,8500 | 1,9700 | 2,0560 | 2,1300 | 2,2158 | 2,4211 | 2,6411 | 2,7020 |  |  |
| PROGESTE | 39 | STD | 24 | 0,1721 | 0,2542 | 0,4004 | 0,2835 | 0,2546 | 0,4198 | 0,4017 | 0,5544 | 0,9312 | 0,8778 | 0,6886 | 0,6843 | 0,6064 | 0,5622 |  |  |
| PROGESTE | 40 | MEAN | 20 | 1,5500 | 1,6071 | 1,6167 | 1,6818 | 1,7135 | 1,8100 | 1,8980 | 2,0188 | 2,1067 | 2,1800 | 2,2675 | 2,4819 | 2,6453 | 2,7012 | 2,8659 |  |
| PROGESTE | 40 | STD | 20 | 0,1301 | 0,2175 | 0,3362 | 0,2321 | 0,3662 | 0,5187 | 0,5771 | 0,5049 | 0,8393 | 0,8785 | 0,7840 | 0,8261 | 0,5898 | 0,6849 | 0,7885 |  |
| PROGESTE | 41 | MEAN | 16 | 1,5546 | 1,5838 | 1,6107 | 1,6792 | 1,7454 | 1,8100 | 1,8864 | 2,0655 | 2,1006 | 2,1714 | 2,3220 | 2,4691 | 2,6975 | 2,7164 | 2,8053 | 2,9827 |
| PROGESTE | 41 | STD | 16 | 0,1303 | 0,3137 | 0,3283 | 0,2841 | 0,3470 | 0,4430 | 0,4806 | 0,6849 | 0,6688 | 0,9158 | 1,0991 | 0,9525 | 0,6021 | 0,7326 | 1,0219 | 0,9218 |
| SO4E1 | 37 | MEAN | 16 | 1,9679 | 2,3143 | 2,2717 | 2,4577 | 2,8362 | 3,3021 | 3,1700 | 3,7350 | 4,8688 | 5,9294 | 7,4000 | 8,0550 |  |  |  |  |
| SO4E1 | 37 | STD | 16 | 0,5774 | 0,7640 | 1,0230 | 0,7891 | 0,6316 | 0,6524 | 0,8180 | 0,8160 | 1,3280 | 0,8286 | 2,3860 | 1,1809 |  |  |  |  |
| SO4E1 | 38 | MEAN | 30 | 1,9304 | 1,8854 | 2,2712 | 2,5796 | 2,4852 | 2,6176 | 3,0448 | 3,1096 | 4,2896 | 6,1904 | 7,1971 | 7,6900 | 13,0010 |  |  |  |
| SO4E1 | 38 | STD | 30 | 0,6322 | 0,6964 | 0,8629 | 0,6570 | 0,9513 | 1,0772 | 1,1274 | 1,1344 | 1,5412 | 3,1075 | 3,8631 | 4,5303 | 7,7600 |  |  |  |
| SO4E1 | 39 | MEAN | 24 | 2,4624 | 2,3870 | 2,5891 | 2,4215 | 2,6447 | 2,9976 | 3,4167 | 4,0183 | 4,3338 | 5,8250 | 7,1563 | 7,5689 | 10,9750 | 15,9727 |  |  |
| SO4E1 | 39 | STD | 24 | 0,7525 | 0,9178 | 0,9759 | 0,8760 | 0,8683 | 0,9201 | 0,9383 | 1,3480 | 1,5449 | 2,2281 | 2,3597 | 1,7734 | 3,3065 | 4,7130 |  |  |
| SO4E1 | 40 | MEAN | 20 | 2,1178 | 2,4611 | 2,5493 | 2,9053 | 3,3547 | 3,5660 | 3,5600 | 4,4529 | 4,2120 | 6,7906 | 9,2675 | 12,2288 | 18,3365 | 21,2917 | 25,4563 |  |
| SO4E1 | 40 | STD | 20 | 0,7032 | 1,3750 | 0,7628 | 0,5884 | 0,7724 | 1,3678 | 1,0684 | 1,7712 | 1,4697 | 2,6359 | 4,0624 | 3,4892 | 3,7562 | 8,6703 | 9,9613 |  |
| SO4E1 | 41 | MEAN | 16 | 2,5907 | 2,5169 | 2,7462 | 3,6246 | 3,4508 | 4,3346 | 4,3977 | 4,8867 | 4,8994 | 6,4693 | 8,5240 | 11,1230 | 17,3909 | 23,2379 | 29,7314 | 37,5687 |
| SO4E1 | 41 | STD | 16 | 1,5913 | 0,5264 | 0,5311 | 0,5386 | 0,4348 | 1,6768 | 0,9180 | 2,1361 | 1,4920 | 2,3069 | 3,0926 | 2,2297 | 2,9989 | 5,0377 | 4,2569 | 5,8905 |
